# Oncology and cardiology positron emission tomography/computed tomography faced with COVID-19: A review of available literature data

**DOI:** 10.3389/fmed.2022.1052921

**Published:** 2022-10-19

**Authors:** Ryogo Minamimoto

**Affiliations:** Division of Nuclear Medicine, National Center for Global Health and Medicine, Tokyo, Japan

**Keywords:** COVID-19, PET/CT, oncology, cardiology, vaccine, FDG

## Abstract

The COVID-19 pandemic has forced people to significantly change their lifestyles and attitudes, and has greatly burdened healthcare delivery systems worldwide. The redistribution of the medical delivery system to maintain normal medical care while responding generously to COVID-19 is a continuing challenge that weighs heavily on medical institutions. Among imaging modalities, chest X-rays and computed tomography (CT) examinations have clearly made a large contribution to treatment of COVID-19. In contrast, it is difficult to express the standpoint of nuclear medicine examinations in a straightforward manner, as the greatest emphasis in this modality has been on how necessary medical care can continue to be provided. Many clinical reports of nuclear medicine examinations related to COVID-19 have been published, and knowledge continues to accumulate. This review provides a summary of the current state of oncology and cardiology positron emission tomography (PET) examinations related to COVID-19, and includes preparation of the nuclear medicine department, trends in PET examinations, specific imaging findings on ^18^F-fluorodeoxyglucose (FDG) PET/CT, imaging of complications of COVID-19, PET tracers other than FDG, and the effects of vaccines on PET imaging findings.

## Publication search

Primary literature was collected from electronic databases PubMed for the positron emission tomography (PET) studies related to COVID-19 until August 10th, 2022, with the search terms: (PET) AND (COVID-19). References cited in the primary literature were manually evaluated for inclusion or exclusion for this review.

## The nuclear medicine department in the setting of COVID-19

Positron emission tomography/computed tomography (PET/CT) is not used routinely in an emergency setting and is not the first choice for diagnosis of infectious diseases. For example, ^18^F-fluorodeoxyglucose (FDG) PET/CT requires the patient to wait for at least 60 min after injection in a confined area, followed by approximately 20 min (depending on the machine) for scanning in the specified examination room. The lengthy period of care required by COVID-19 patients in a small and closed space with limited equipment is a burden for the staff of a nuclear medicine (NM) department, and also risks interaction between patients. Moreover, incidental findings of possible COVID-19 infection in patients having scheduled or ongoing PET/CT scans disrupts the workflow of the department. During the COVID-19 pandemic, the role expected of the NM department was to continue to provide essential and critical services. In advanced preparations, the NM department was required to establish effective procedures for patient and staff flow when facing known, suspected, and incidentally detected COVID-19 patients, and should control transmission of the virus while continuing to provide essential and critical services (1). In addition, the NM department was expected to maintain education, research, and the conduct of clinical trials as much as possible during the pandemic; therefore, appropriate workflow should be established to be able to cover all tasks assigned to the department (2, 3). Accordingly, it was essential to develop contingency and business continuity plans for operation during the emergency state.

Several guidance documents and guidelines for NM departments were announced during the COVID-19 pandemic, which were generally based on the six main processes outlined in the World Health Organization (WHO) guidelines, “COVID-19: Operational Guidance for maintaining essential health services during an outbreak” (4). Staff working in the NM department were required to receive specific training in identifying COVID-19 symptoms, social distancing, hygiene control, handling COVID-19 patients, disinfection procedures, and maintaining the availability of essential equipment and supplies such as personal protective equipment. The use of communication technologies for teleconsultation and remote reporting was considered an effective way to deal with this situation (1, 5, 6).

Boscombe et al. (7) proposed the traffic light system for decision-making in scheduling NM examinations. Examinations in the “Green” category could be rebooked without discussion with a clinician, and no PET examinations were included in this category. Those in the “Amber” category could be discussed with a clinician if there was a need to cancel/rebook, and the applicable categories were follow-up PET examination using FDG, ^68^Ga-DOTATATE, ^68^Ga-PSMA, and ^18^F-choline (FCH). Examinations in the “Red” category could not be cancelled or rebooked unless the patient was at high risk, and the applicable categories were FDG assessment for new cancer and sepsis, ^68^Ga-DOTATATE for staging and therapy decision, and ^68^Ga-PSMA for new cancer (7).

Early in the pandemic, our department reported a preparatory protocol for PET/CT examination of COVID-19-infected patients (8). The major points considered were pre-checking of the patient clinical record; patient transportation, preparation of equipment for staff, the examination room and the patient waiting room; and zoning of the department floor, arrangement of staff, method of conducting patient care; and cleaning and recovery of the examination room. The protocol was designed based on the abundance of accumulated knowledge at that time, and was continuously updated according to emerging evidence (8).

In an international survey of the circumstances of NM departments that was conducted early in the COVID-19 pandemic, approximately half continued to function but reduced the number of NM examinations and therapy sessions; 46% of facilities allowed only urgent NM examinations, and just 3% of facilities did not modify their schedule. A decrease of more than 50% of NM examinations was confirmed in 44% of NM departments. The most affected diagnostic NM examinations were cardiology (26%), followed by oncology (20%), neurology (19%), endocrinology (14%), urology (11%), and infection/inflammation (8%). PET (11%) and radionuclide therapy (19%) procedures were less affected than scintigraphy/single photon emission computed tomography (SPECT) (70%) (9).

It was also necessary to consider transition of the NM department back to normal operation, with the expectation that there would be a shift to “new normal” operation. Huang et al. (10) proposed a step-wise reopening schedule (traffic light system) for a large NM department. Key factors in the decision to relax the restrictions placed in response to COVID-19 were the priority of the examination, patient capacity, availability of essential materials, human resources, and having the necessary assistive technology (10).

## Impact of COVID-19 on positron emission tomography/computed tomography examinations

Across 96 countries, the volume of PET examinations decreased by 36.0% in April 2020, 65.6% in June 2020 and 40.3% in October 2020 compared to the average number of procedures before the COVID-19 pandemic. The decline in utilization was less for oncological PET examinations than for conventional NM examinations. The impact was significantly pronounced in Latin America, South East Asian countries, lower-middle -income countries. A gradual return to the pre-COVID-19 situation of supply chains for radioisotopes, generators, and other essential materials has been confirmed (11). A survey conducted in 72 countries in April 2020 revealed that PET/CT scans decreased by an average of 36%, but were less affected than conventional NM, which showed decreases for lymph node (LN) procedures by 45%, lung scans by 56%, bone scans by 60%, myocardial studies by 66%, and thyroid studies by 67%. Insufficient supplies of essential materials (radioisotopes, generators, and kits) were reported, especially for ^99m^Tc/^99^Mo generators and ^131^I, particularly in Africa, South Asia, and Latin America (12).

Compared to a pre-COVID-19 baseline, the utilization of PET procedures in Africa (16 countries) decreased by 58% in June 2020 and by 45% in October 2020. Latin America had the largest decrease, by 89% in June and 48% in October 2020. Compared to conventional NM, PET examinations were less affected in Africa and more affected in Latin America in June, but recovered in October 2020 despite the confirmed 45–48% reduction in PET examinations (13). At Massachusetts General Hospital and 26 affiliated imaging centers between 1 January 2020 and 21 May 2020, imaging volume drastically decreased after 11 March 2020 as non-essential imaging examinations were postponed and non-essential in-person activities were deferred in response to the declaration of a state of emergency in Massachusetts. The NM imaging volume decreased by 78.3% after declaration of the state of emergency. On 17 May 2020, the rate of decrease was still 69% for NM, which was a much slower recovery of imaging numbers compared with CT, MRI, US, and radiography (14). In another study, NM (61% reduction) and mammography (93% reduction) were the most affected modalities after 10th March 2020 in northeast Ohio (including the Cleveland metropolitan area and its surrounding counties) (15).

Norbash et al. (16) reported radiology volume in the early stage of the COVID-19 pandemic in facilities with contrasting examination volumes: in three high-surge academic medical systems (AMSs), three low-surge AMSs, and in a coalition of private radiology practices (Strategic Radiology). Steep drops in volume occurred during week 11 in 2020, followed by a slow recovery from week 17. Compared to the PET/CT imaging volume in 2019, volume dropped to 33% of baseline in the high-surge AMSs. Volume dropped to 84% of baseline in the low-surge AMSs, which was the least decrease in volume for any modality. The authors concluded that the trend in the volume of PET/CT was partly related to the high volume of cancer patients undergoing PET/CT examinations, and also to patient intolerance of delays in care, including delayed PET/CT imaging, that could potentially influence their survival (16).

According to a survey on the impact of the first wave of COVID-19 (estimated to have started in March 2020 and ended at the end of May 2020) on NHS England PET/CT services, there was a reduction in the number of FDG PET/CT examinations of 32% in April 2020 and 31% in May 2020 compared with those performed in the same months in 2019, and recovered to a reduction of 6% in June 2020. The first wave of COVID-19 had a much greater influence on non-oncological FDG PET/CT (reduction of 55% in April 2020 and 33% in May 2020) than on oncological PET/CT (reduction of 23% in April 2020 and 26% in May 2020) (17), similar to the trends reported in other studies (12, 18). This finding was the result of recommendations by professional organizations and published guidance that PET/CT services for people with cancer should remain uninterrupted as far as possible (1). In April 2020, the percentage decrease was remarkable for esophageal cancer and lung cancer, whereas the smallest decrease was for melanoma and malignant lymphoma (ML), which then increased in May and June 2020 (17). In January 2021, the number of PET examinations remained stable (69%) in the majority of 32 European countries; in the remainder, the decrease was generally less than 25%. Oncological FDG-PET was the most severely affected examination (19). Maurea et al. (20) reported trends in the number of FDG PET/CT examinations performed at a single medical institution in Italy during three COVID-19 waves: (1) 3 February to 30 April 2020, (2) 15 October 2020 to 15 January 2021, and (3) 18 January to 16 April 2021. There was no change in the number of FDG PET/CT examinations during the three waves, but the number of patients with COVID-19 infection increased with each successive wave (20). These findings indicate that the number of PET examinations was temporally affected after declaration of the COVID-19 pandemic, but normality returned reasonably quickly.

## ^18^F-fluorodeoxyglucose positron emission tomography/computed tomography imaging findings in COVID-19 patients

### COVID-19 pneumonia

Patients with cancer and cardiovascular disease have a greater risk for worse clinical outcomes with COVID-19 infection (21). The risk of developing severe events in COVID-19 is statistically significantly higher in patients with cancer, with a hazard ratio of 3.56 (22). The incidence of positive CT findings specific to COVID-19 was high among those who were asymptomatic but were positive by reverse transcription-polymerase chain reaction (RT-PCR) testing (23). Chest CT has been crucial for identifying COVID-19 pneumonia, which has typical findings of bilateral lung involvement, predominantly in peripheral, subpleural, and posterior areas of the lung, with ground-glass opacities (GGOs) with or without consolidations, linear opacities and crazy-paving pattern (24–27).

**FIGURE 1:**
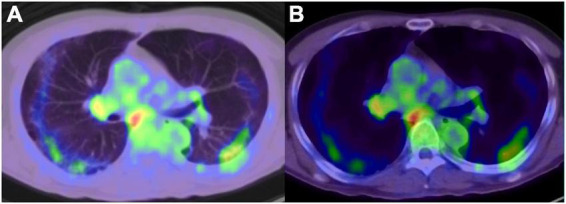
^18^F-fluorodeoxyglucose -positron emission tomography/computed tomography image of COVID-19 pneumonia, and reactive mediastinum and hilar lymph node. **(A)** Lung window, **(B)** mediastinal window.

Numerous studies have reported the incidental detection of COVID-19 infection at the time of FDG PET/CT examination in patients with or without malignancy (28–32). SARS-CoV-2 infects cells expressing the surface receptors angiotensin-converting enzyme 2 (ACE2) and transmembrane protease serine 2 (TMPRSS2). The active replication and release of the virus lead the host cell to undergo pyroptosis and release damage-associated molecular patterns. These patterns are recognized by neighboring epithelial cells, endothelial cells and alveolar macrophages, and trigger the generation of pro-inflammatory cytokines and chemokines (33). Thus, FDG uptake in segmental ground-glass density lesions suggests a high level of inflammatory-related processes occurring in the lesions, even if the CT findings indicate an early stage of COVID-19 infection (Figure 1).

Between 16 and 24 March 2020, 6/65 patients (9.2%) who underwent PET/CT at a single institution in Italy for assessment of malignancy showed unexpected signs of interstitial pneumonia on CT and elevated regional FDG uptake (30). Similarly, another study reported that suspected interstitial pneumonia due to COVID-19 infection was significantly more frequent (9%) during the pandemic (between February and April 2020) than in the same months in 2019 (4%) (18). In Italy, which had a high prevalence of COVID-19, the rate of interstitial pneumonia suspected to be caused by COVID-19 infection was significantly higher during a COVID wave (7.1% in 16–27 March 2020) than in pre-COVID (5.35% in January–February 2020), and control periods (5.15% in 2019) (34). In Nantes, France, COVID-19 infection was detected incidentally in 3.8% of FDG PET/CT examinations during the pandemic (March to April 2020) than before the outbreak of COVID-19 (Jan to Feb 2020, 2.2%) (35). In the UK, the ratio of incidental findings on FDG PET/CT imaging in spring 2020 (16.3%) showed no difference from that in spring 2019 (16.1%); however, the incidence increased significantly with time in 2020 [2nd week, odds ratio (OR) = 3.8; 3rd week, OR = 7.6, compared to the 1st week] (36). The changing trend of incidental findings on FDG PET/CT may have been affected by the reference period and the surveyed region. However, it was clear that COVID-19 pneumonia could develop in asymptomatic patients, some of whom were incidentally diagnosed at a scheduled FDG PET/CT examination.

^18^F-fluorodeoxyglucose PET/CT could detect lung infiltrates from COVID-19 in asymptomatic patients at a mean of 6 days (range, 1–24 days) prior to symptom onset (37). It is crucial following FDG PET/CT examination that the CT component of PET/CT is carefully reviewed on lung window settings before the patient leaves the scanner. By checking for abnormal signs, an early decision can be made regarding COVID-19 infection, and the doctor in charge can be alerted immediately. This is particularly important in patients in an immunosuppressed state due to anticancer treatment or surgery, and alerts staff that the examination room needs to be thoroughly cleaned before the next patient is scanned (1).

**FIGURE 2:**
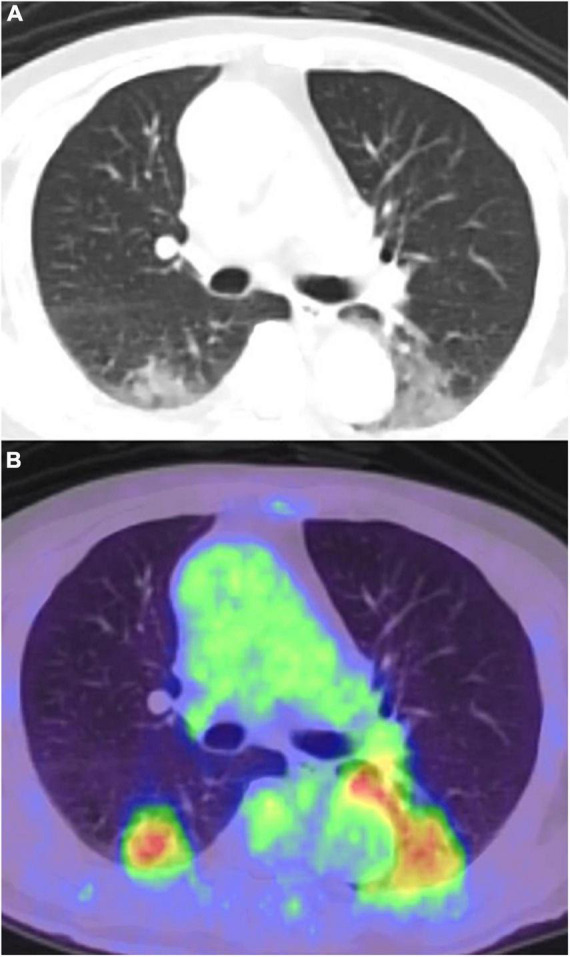
Pneumonia caused by bronchoscopy performed a day before the FDG-PET/CT, misidentified as COVID-19 pneumonia. **(A)** CT image, **(B)** PET/CT image.

The differential diagnoses of COVID-19 pneumonia are infectious disease; non-tuberculous mycobacterial infections; and non-infectious diseases such as pulmonary edema, hemorrhage, neoplasms, organizing pneumonia, pulmonary alveolar proteinosis, sarcoidosis, pulmonary infarction, interstitial lung diseases, and aspiration pneumonia (38, 39) (Figure 2). However, it is difficult to distinguish COVID-19 pneumonia from these diseases by FDG PET/CT imaging alone.

In a review of 18 studies of 30 asymptomatic patients (mean age, 62 ± 15 years) with COVID-19 who underwent FDG PET/CT, lung lesion patterns specific to COVID-19 pneumonia were found in 93% of patients and FDG-avid lung lesions were found in 90%. The lung lesion pattern was GGO with other lesion patterns in 50% of patients and GGO alone in 43%, and the lesions tended to occur at multiple sites (70%), in more than two lobes (60%), and in both lungs (70%). The maximum standardized uptake value (SUV_max_) in lung lesions with FDG uptake ranged from 2 to 12 (40). In another study, the common PET/CT findings were hypermetabolic bilateral GGOs (75%), consolidation (35%), and interlobular thickening (8%) in 52 patients (mean age, 60 ± 13 years) with COVID-19 infection (confirmed by RT-PCR test using nasopharyngeal swabs) and at least one PET/CT examination (FDG, 92.3%; FCH, 5.8%; and ^68^Ga-PSMA, 1.9%). The mean SUV_max_ for pulmonary lesions with FDG uptake was 4.9 ± 2.3 (range, 1.2–18) (41).

No significant correlation was found between PET/CT findings (lung SUV_max_, lung hypermetabolic volumes, and mediastinal LNs SUV_max_) and chest CT evolution, or C-reactive protein (CRP). Moreover, PET lung inflammatory status showed no correlation with short-term clinical outcomes of patients with COVID-19 (42). Yeh et al. reported that lung SUV_max_ of FDG uptake was not associated with COVID-19 symptoms, severity, or disease course, but that a positive PET scan was associated with higher risk of symptomatic infection and hospitalizations despite the limitations of FDG PET/CT for detection of COVID-19 infection (41.9%) (43).

In contrast, Triviño-Ibáñez et al. (44) reported that volumetric FDG PET/CT measurement results (SUV_peak_ and pulmonary total lesion glycolysis [TLG]) were correlated with laboratory and respiratory parameters in the short-term follow-up of patients hospitalized for COVID-19 pneumonia. SUV_peak_ for a target lesion in the mediastinum was correlated with% predicted diffusing capacity of the lungs for carbon monoxide (DLCO) (ρ = 0.782), CO transfer coefficient (KCO) (ρ = 0.721), and residual volume (RV) (ρ = 0.636). Pulmonary TLG was significantly and negatively correlated with% predicted DLCO (ρ = –0.628), KCO (ρ = –0.564), total lung capacity (ρ = –0.532), and RV (ρ = –0.554) values (44).

The increase of FDG uptake in lung lesions with time indicates increasing lung inflammation in the acute stage of COVID-19 infection. COVID-19 Reporting and Data System (CO-RADS) criteria applied to CT findings showed a relation to FDG uptake (SUV_max_) in the lung parenchyma in asymptomatic cancer patients (45). Thornton et al. (46) showed that a lung target-to-background ratio (TBRlung [SUV_max_/SUV_min_]) was strongly correlated with time after infection within 3 weeks after infection, and was higher in the late stage of COVID-19 infection (>1 week to ≤4 weeks after disease onset) than those in the early stage (approximately ≤ 1 week after disease onset) (TBRlung 6.4 vs. 13.7). TBRlung was lower in asymptomatic recovered patients (4.6) than in either untreated post–COVID-19 lung disease patients (18.1) or those treated with steroids (6.62) (46).

In general, FDG uptake generally decreases with viral clearance and the establishment of immunity. However, an FDG PET/CT study revealed that significant inflammation remained in the lungs, mediastinal LNs, spleen, and liver after two consecutive negative RT-PCR tests in patients recovering from severe COVID-19 infection (47, 48). The dissociation between recovery according to CT and delayed recovery of FDG uptake in COVID-19 lesions indicated that a high level of inflammatory change persisted even in the recovery stage with an activated host immune response and/or angiovascular damage (46, 48–50).

## ^18^F-fluorodeoxyglucose uptake in lymph nodes of patients with COVID-19

^18^F-fluorodeoxyglucose uptake in normal-sized LNs is a common observation in patients with COVID-19 and is thought to indicate immunoreactions activated by inflammatory cells such as neutrophils, monocytes, and effector T cells by the release of local chemokines. In the immune response to viral infections, the number of monocytes in lymphoid tissue increases, leading to increased FDG uptake (51, 52).

Lymph node enlargement is a rare finding on CT, and is reported to occur in <1% of patients with COVID-19 (53). LNs with intense FDG uptake are generally small, non-specific, and regular in shape (52). In another report, FDG uptake was confirmed in mediastinal LNs without significant enlargement, and the uptake decreased during 4 weeks of observation. CT showed little change in LN size during the clinical course, but CT may be less sensitive to host reactions compared with FDG PET/CT, and therefore the actual percentage of LN involvement may be higher than seen on CT (50).

^18^F-fluorodeoxyglucose uptake after COVID-19 infection was seen most frequently in mediastinal LNs (27%), followed by hilar (19%), subcarinal (10%), subclavian (6%), paratracheal (6%), thoracic (4%), and subdiaphragmatic (4%) LNs (41). The SUV_max_ of thoracic LNs ranged from 2.5 to 9.6 (40).

Several studies have reported that negative FDG uptake may occur in these LNs in the minimally invasive and early stages of the disease (54). The immune response is weak or almost absent in the early stage and becomes more active over time. Moreover, reduction of FDG uptake in LNs may indicate normalization of hyperactive immune response in the body.

## Additional findings on ^18^F-fluorodeoxyglucose positron emission tomography/computed tomography during and after COVID-19 infection

COVID-19 can induce immune-related manifestations with systemic and organ-specific disorders, which are associated with excessive immune response caused by systemic excessive cytokine production (55). Immune response disorder related to COVID-19 has been reported in the kidneys as well as the pulmonary, cardiovascular, neurological, gastrointestinal (GI), and hepatobiliary systems (56–58). Bai et al. (47) reported that FDG uptake in liver (SUV_max_ and SUV_avg_) and spleen (SUV_max_) was significantly higher in COVID-19 patients than in healthy controls, whereas there was no difference in uptake in the left ventricular lateral wall, small intestine, and renal cortex. FDG uptake in the spleen was significantly correlated with blood lymphocyte count (*r* = 0.80–0.86) (47). Dietz et al. confirmed FDG uptake in the spleen (38%), bone marrow (15%), and nasopharynx (23%) in COVID-19 patients (42). Uptake of FDG in the bone marrow and/or spleen has been confirmed in COVID-19 patients in several other studies (42, 50, 59–62). In patients with COVID-19, neutrophils are more abundant and scattered plasma cell infiltration is more frequent in the spleen. It has been suggested that pathological changes in the spleen might be related to direct attack by the virus and immune cells (63).

Halsey et al. reported extra-thoracic findings of increased FDG uptake in the tonsils, salivary glands, and small and large bowel on FDG PET/CT in 7% of the study group (36). In patients with COVID-19 syndrome of moderate severity, higher FDG uptake was observed in the ileum, cecum, and colon; and that in the cecum remained at recovery. Comparing FDG uptake with lymphocyte subsets, CD3+/CD4+/CD45+ significantly correlated with FDG uptake in the cecum and colon, and CD3+/CD8+/CD45+ significantly correlated with that in the lungs and bone marrow (62).

Thrombotic complications are a frequent occurrence in patients with severe COVID-19. The incidence of venous thromboembolism and arterial thrombotic event have been reported as 27 and 3.7%, respectively (64). FDG PET/CT identified incidental pulmonary embolism in 2/23 (14.3%) patients with pulmonary infiltrates suggestive of COVID-19 (65).

The FDG PET/CT pattern differs between infection with the Omicron variant and that with earlier variants in terms of symmetric FDG uptake at the nasopharynx, oropharynx, and tonsils, with or without associated FDG-avid cervical lymphadenopathy, particularly in the suprahyoid neck (66).

Sollini et al. (67) used FDG PET/CT to evaluate the persistent inflammatory process in recovered adult COVID-19 patients who complained of unexplained persisting symptoms lasting more than 30 days. In the COVID-19 group, FDG uptake in different regions of the aorta (ascending, aortic arch, and descending) and in the right iliac artery, and the femoral artery-to-blood pool ratio were statistically significantly higher than those of the control group. They concluded that the vascular inflammation induced by COVID-19 may be responsible for the persistent symptoms (67).

The COVID study aimed to assess the presence of aortic inflammation and its time-dependent trend in patients with COVID-19, and found no significant difference in aortic FDG PET/CT uptake between COVID-19 patients and controls (global aortic target to background ratio [GLA-TBR], 1.46 vs. 1.43, respectively). However, GLA-TBR was moderately associated with high sensitivity CRP and with days from admission to FDG PET/CT in COVID-19 patients. Index aortic segment TBR (IASTBR) also showed a moderate association with high sensitivity CRP and days from admission to FDG PET/CT. IASTBR was significantly higher in patients scanned ≤60 days from admission than in controls, and recovered at the same level as controls at >60 days after admission for COVID-19 (68). In another report, segmental FDG uptake at the abdominal aorta was confirmed in COVID-19 patients in the early phase, and showed remission without immunosuppressive treatment 30 days later (69).

An FDG PET/CT study of adult patients with long COVID, defined as at least one persistent symptom for more than 30 days after recovery from infection, reported uptake in post pneumonia lung abnormalities, bone marrow, vascular system, joints, and several organs along with hypometabolism in the right parahippocampal gyrus and thalamus (70). As COVID-19 can inflict damage on the entire body, total body FDG PET imaging may contribute to evaluating the extent of the disease and quantifying its severity in various organs (71). Although no absolute necessity has emerged for FDG PET/CT in the diagnosis or management of COVID-19, it may nevertheless offer insight into the background of patients who show atypical symptoms after COVID-19 infection and long-term effects as called “Long COVID” (72). In this regard, research that utilizes neurological molecular imaging is continuing to advance (73).

## COVID-19 vaccination

The Centers for Disease Control and Prevention (CDC) publicly notified that the incidence of lymphadenopathy was higher after the Pfizer-BioNTech COVID-19 vaccination (*n* = 64) compared with a placebo group (*n* = 6). Although lymphadenopathy was defined as an unsolicited adverse event in this clinical trial, lymphadenopathy in the arm and neck regions was confirmed within 2–4 days after vaccination and the average duration of lymphadenopathy was approximately 10 days (74). In a Moderna clinical trial with a cohort aged 18–64 years, axillary swelling or tenderness was regarded as a solicited adverse event that occurred in 11.6% of patients after the first vaccination and in 16.0% after the second vaccination, which was higher than the incidence with placebos (5.0 and 4.3%, respectively) (74). This reaction was less common in subjects aged ≥ 65 years, occurring in 8.4% of this group after the second dose (74, 75). These two mRNA vaccines appear to stimulate immune activity to a greater degree than vaccines based on traditional biotechnologies (76). During the period covered by the above clinical trial, the size and extent of lymphadenopathy enabled ready identification by palpation and/or visual inspection. In fact, LN size varies after vaccination, from normal to moderately increased, with benign features such as thickening of the cortex and fatty hilum. However, LNs can show abnormal size and loss of fatty hilum shortly after vaccination, which can be potentially misinterpreted as malignancy.

The immune response increases glucose metabolism in lymphoid organs, which are critical modulators of T-cell immunity (77). FDG uptake in small axillary LNs (ALNs) is a well-known feature after vaccination against influenza (54, 78–81) and other diseases (82).

**FIGURE 3:**
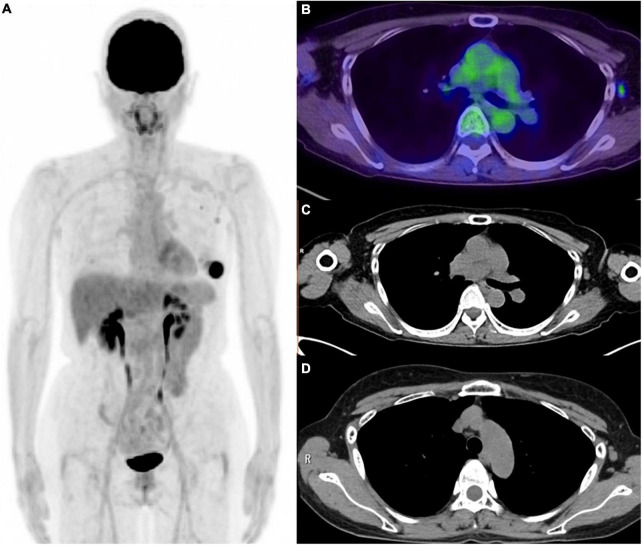
Six weeks after the COVID-19 vaccination for the patient with left breast cancer. **(A)** MIP image of FDG PET, **(B)** axial image of PET/CT, **(C)** CT portion of PET/CT, **(D)** CT image (2 weeks after the vaccination). Weak FDG uptakes were confirmed in the small axial lymph node and subclavian lymph node. These lymph node was pathologically diagnosed as lymph node metastasis from left breast cancer. Axial lymph node in CT image obtained 2 weeks after the vaccination was slightly larger than those in the PET/CT image, which might be the effect of vaccination.

Compared to traditional vaccines, the higher body response to the COVID-19 vaccines is confirmed in FDG-PET imaging (51). FDG uptake has been identified in normal-sized to moderately enlarged axillary supraclavicular and cervical area nodes following intramuscular vaccination in the ipsilateral deltoid (83–97). FDG uptake in LN is associated with immune system activation, whereas FDG uptake in deltoid muscle is caused by inflammatory etiology or trauma induced by the injection (88). LNs on the injected side are mostly affected, but contralateral LNs can also show FDG uptake (97). Therefore, the FDG PET/CT findings can lead to misdiagnosis in the evaluation of malignancy and inflammatory disease, particularly with regard to breast cancer, melanoma (trunk or upper extremity), sarcoma, ML, lung cancer (particularly upper lobe), head and neck cancer, Castleman disease, and sarcoidosis (95) (Figure 3). FDG PET/CT should not be postponed in the case of recent vaccination if the patient has an urgent or pressing need, such as requiring disease staging or treatment initiation. However, as FDG PET/CT findings caused by vaccination can possibly lead to misdiagnosis, such patients should be followed up closely with further investigations performed as necessary. In particular, a clinically relevant finding such as morphological abnormality should be assessed by US or CT in 2–6 weeks and US-guided sampling can be considered if the abnormal nodes persist at that time (95). This recommendation is supported by the finding that vaccine-related lymphadenopathy is more likely to occur in level II and III nodes than in metastatic nodes from breast cancer, in which lymphadenopathy is most common in level I/II nodes (lower part) (98).

Urgent serial vaccination was recommended as the COVID-19 pandemic spread, and as the trend in the number of COVID-19 antibodies over time became apparent. This led to the dilemma of not being allowed to delay either vaccine administration or FDG PET/CT examination. It is unknown whether COVID-19 will become a seasonal trend that requires continued and regular vaccination. It appears that the schedule adjustment of FDG PET/CT and vaccination will be kept required in the future, but it may be given a more sufficient period of time for the scheduling than now.

^18^F-fluorodeoxyglucose -avid lymphadenopathy reported in numerous studies ranged from 13 to 90% in patients after vaccination for COVID-19 (86–88, 90, 96, 99–102), and the pooled value was 37% (95%CI, 27–47) (103) even with significant heterogeneity among the studies. Cohen et al. categorized vaccine-associated hypermetabolic lymphadenopathy (VAHL) according to the intensity and area of FDG uptake in axial LNs as follows: grade 1, mild FDG-uptake intensity (SUV_max_ < 2.2); grade 2, moderate FDG-uptake intensity (2.2 ≤ SUV_max_ < 4); grade 3, high FDG-uptake intensity (SUV_max_ ≥ 4) in normal-size nodes; and grade 4, high FDG-uptake intensity (SUV_max_ ≥ 4) in enlarged nodes. The incidence of VAHL was 36.5% among the entire sample of vaccinated subjects, and was significantly higher after the second vaccination (45.8%) than after the first vaccination (26.3%). Regarding the first vaccination, the incidence of VAHL was higher at 6–12 days after vaccination compared with that in the first 5 days and at 13 days or more. In the second vaccination, the incidence and grade of VAHL were highest in the first 6 days, and decreased gradually over time to become significantly low at >20 days after vaccination. VAHL was confirmed in 29% of vaccinated patients within 3 weeks after the second vaccination, but only 7% had grade 3 or 4 VAHL. After the first vaccination, there was a higher incidence and higher grade of VAHL in subjects aged ≤ 62 years than in others; whereas higher incidence and higher grade of VAHL were confirmed in subjects aged ≤ 64 years than in others after the second vaccination (90).

Su reported that FDG uptake in ALNs due to the COVID-19 vaccine was the most intense in the first two weeks after vaccination and decreased over time. Approximately half of the patients demonstrated low-grade FDG uptake compared to background in ALNs until 5–6 weeks after vaccination (104). Skawran et al. (102) reported that PET/CT showed FDG-avid ALNs ipsilateral to the vaccine injection site in 54% of 140 oncological patients after COVID-19 vaccination, and uptake was still present in 38% of patients at 28 days after vaccination. FDG avidity was more common in the LNs of patients vaccinated with the Moderna vaccine (72%) than with the Pfizer-BioNTech vaccine (43%) (102).

Advani et al. (105) showed the time course of FDG uptake in the ipsilateral axillary/sub-pectoral LNs. FDG uptake was confirmed in 70% of subjects 0–7 days after receiving the vaccine, and dropped to 55% after 8–14 days, and to 44% after 2 weeks. However, it did not fall below 40% after 4 weeks (105). Other studies have reported persistent FDG uptake in the ALNs at 4–6 weeks (83) and at 7–10 weeks (87) after injection.

After the third COVID-19 vaccination, the incidence of all-grade VAHL and of grade 3–4 VAHL was reported as 47.5 and 8.9%, respectively. VAHL was identified in 82.5% of FDG PET/CT studies performed within the first 5 days after Pfizer-BioNTech vaccination (106). A third vaccination seemed to have reduced effects on FDG PET/CT imaging results compared to the first and second vaccinations.

The trend of the effect of clinical background on FDG uptake after vaccination has been assessed in several studies (Table 1). A strong inverse association was confirmed between positive FDG uptake in ipsilateral LNs and patient age (OR, 0.57), immunosuppressive treatment (OR, 0.37), and presence of hematologic disease (OR, 0.44); whereas the scaled number of days from the last vaccination (OR, 1.53) and the second vaccination (OR, 7.53) showed a positive association with positive FDG uptake in ipsilateral LNs. Age and sex were not related to uptake. No index has shown any association with FDG uptake in deltoid muscle (82). El-Sayed et al. (107) reported that women were more likely to have reactive ALN, and that the frequency and intensity were higher in patients aged < 65 years. The trend did not differ by vaccine type in this study cohort (107). FDG-positive ipsilateral ALNs were confirmed until 8–10 weeks after vaccine injection (<2 weeks, 63%; 2–4 weeks, 42%; 4–6 weeks, 26%; 6–8 weeks, 15%; and 8–10 weeks, 19%).

**TABLE 1:** Significant factors related to the FDG uptake in ALN after COVID-19 vaccination.

Author	Number of subject	Vaccine types	Significant factors related to the FDG uptake in ALN				
			Age	Sex	Days after the last vaccine injection	First or second vaccination	Others
Kubota K	202	Pfizer-BioNTech	Negative (mean 74 years old) >Positive (mean 69 years old)	W (52%) > M (35%)	negative case (mean 15 days) > positive (mean 10 days)	Second (51%) > First (41%)	
Eifer M	426 (FDG: 377, other tracers: 49)	Pfizer-BioNTech	OR 0.57	ns	OR 1.53	Second (OR 7.53)	With Immunosuppressive treatment (OR 0.37), with hematologic disease (OR 0.44)
El-Sayed MS	204	Pfizer-BioNTech or Oxford-AstraZeneca	<65 years old	W (51%) > M (35%) less than 6 weeks post vaccination	–	–	Oxford-AstraZeneca (53%) > Pfizer-BioNTech (33%) less than 6 weeks post vaccination
Seban RD	260 (of 233 received vaccine)	–	=50 years old (OR 2.4)	–	<30 days (OR 2.3)	ns	Without lymphopenia (OR 1.9)

ALN, axillary lymph node; OR, odds ratio.

Kubota et al. (108) reported short duration from vaccination, younger age, female sex, and smaller area of pathological FDG uptake related to the patient’s primary disease as factors related to positive FDG uptake in ALNs. In the first COVID-19 vaccination, there was high FDG uptake in the ALN within 10 days from the injection, which decreased with time but remained in 7.7% of subjects at 30 days after injection (0–4 days, 61%; 5–9 days, 51%; 10–14 days, 36%; 15–19 days, 42%; 20–24 days, 24%; 25–29 days, 22%). FDG uptake in deltoid muscle (the injection site) was common within 4 days of vaccination and diminished after 20 days from vaccination (0–4 days, 61%; 5–9 days, 31%; 10–14 days, 9%; 15–19 days, 3%). Compared with the first vaccination, the occurrence of FDG uptake in both ALN and deltoid muscle was higher after the second vaccination but the duration was shorter. Significant contributors to positive FDG uptake in deltoid muscle were short duration since vaccination and female sex (108). Seban et al. (109) surveyed the incidence of vaccine-induced hypermetabolic LNs (v-HLN) and related factors in patients receiving FDG PET/CT. v-HLN was confirmed in 35% of patients with a median SUV_max_ of 3.7 (range, 2.0–26.3). Age ≤ 50 years (OR, 2.2), absence of lymphopenia (OR, 2.2) and an interval < 30 days between injection of the last vaccine and FDG PET/CT (OR, 2.6) were independent factors for v-HLN (109). Patients with ML who had been treated with an anti-CD20-antibody-containing regimen in the 12 months before vaccination had significantly lower rates of VAHL compared with all other patients with ML (8.8% versus 41.2%). In this cohort, VAHL was confirmed as statistically significantly higher in patients with high anti-spike titers (72%) compared to those with negative serology (10%) and low anti-spike titers (31%). It was estimated that VAHL on FDG PET/CT of patients with hematologic malignancy may reflect prominent B cell germinal-center cell proliferation and an effective humoral response elicited by the BNT162b2 vaccine (99).

## COVID-19 vaccine-related inflammatory response

**FIGURE 4:**
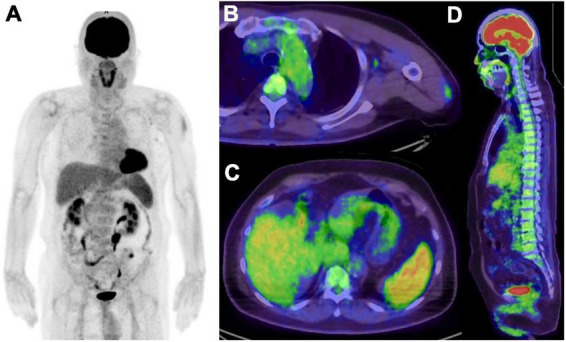
Five days after COVID-19 vaccination in patient suspected recurrence of lung cancer. **(A)** MIP image of FDG PET, **(B,C)** axial image of PET/CT, **(D)** sagittal image of PET/CT. Increased FDG uptake were seen in left deltoid muscle, left axial lymph node, spleen and bone marrow, and all of them related to the vaccination.

As well as COVID-19 infection, COVID-19 vaccination may also induce immune response disorder related to COVID-19 (110, 111). The dominant theory is that vaccine-associated adverse reactions are caused by immunostimulatory and inflammatory cytokine release, autoimmunity, eosinophilia, and ACE2 downregulation (111). Splenic FDG uptake has been reported in addition to ALN uptake at 5 days after COVID-19 vaccination (112) (Figure 4). Another study reported FDG uptake in thymic hyperplasia and in ALN at 10 days after COVID-19 vaccination in patients with mantle cell lymphoma, which the author suggested occurred as an immunologic response to vaccination (113).

In the patients who met the criteria for systemic immune response syndrome (SIRS) within 1 day of the first dose of the COVID-19 vaccine, FDG PET/CT showed focal uptake in the right deltoid area, multiple right ALNs, and diffuse uptake in the spleen. These findings are characteristic of SIRS after COVID-19 vaccination (114). The same appearance with the addition of diffuse FDG uptake in bone marrow was reported in patients with SIRS, but these findings had all disappeared 3 months later (115).

COVID-19 vaccine-induced inflammation has been depicted by PET/CT imaging as mediastinitis, pleuritis, pericarditis, and peritonitis polyserositis (116). Systemic vasculitis following COVID-19 vaccination has also been depicted by FDG PET/CT. Unlike large vessel vasculitis, FDG uptake was confirmed as extensive linear and patchy hypermetabolic foci along middle-sized major arteries and their branches. Pathological findings in the temporal artery showed FDG uptake in markedly destroyed vascular wall structure without giant cell formation (117). Schierz et al. (118) reported a case in which the FDG PET/CT findings indicated vasculitis and bursitis after COVID-19 vaccination. The location of intense FDG uptake in the vertebral artery correlated with arterial wall thickening confirmed by CECT (118). FDG uptake was confirmed in the pericardium along with cardiophrenic lymphadenopathies in patients diagnosed with adult-onset Still’s disease after vaccination with a COVID-19 adenoviral vector vaccine (119). FDG uptake was observed around the shoulder, hip joint and scapulae mimicking polymyalgia rheumatica after COVID-19 vaccination (120).

COVID-19 vaccination was reported to induce FDG-avid multi-station lymphadenopathy and radiation recall pneumonitis, which can be misdiagnosed as recurrence of lesions (121). However, it remains unknown whether COVID-19 vaccination directly induces autoimmune disorders, which may be caused by a genetic predisposition to develop such diseases or may have incidentally occurred simultaneously. It has been advocated that in patients with autoimmune disease, the benefits of COVID-19 vaccination outweigh the risks of worsening autoimmune disease and reduce the risk of severity after COVID-19 infection (122).

## Non-^18^F-fluorodeoxyglucose positron emission tomography tracers for COVID-19

Increased PET tracer uptake in COVID-19 pneumonia has been reported in ^68^Ga-PSMA (123–129), ^18^F-PSMA-1007 (130) ^18^F-PSMA (131), FCH (123, 132–135), ^68^Ga-DOTATATE (124), ^68^Ga-DOTANOC (133), ^68^Ga-FAPI-46 (134), ^18^F-avb6-BP (136), and ^82^Rb (137). ^68^Ga-DOTANOC (130), FCH (123, 132), and ^68^Ga-PSMA (136) uptake has been reported in the mediastinum and hilar LN, suggesting a reactive response. In addition, tracheitis was diagnosed with ^68^Ga-PSMA PET/CT (138). The uptake of PSMA ligand is hypothesized to occur as a result of neovascularization and increased regional blood flow/vascular permeability, leading to the delivery of more PSMA ligand to the inflammation/infection site (139). In addition, folate receptors overexpressed on activated macrophages may interfere with the expression of folate hydrolase/PSMA (123). ^68^Ga-DOTA uptake may originate from somatostatin receptors expressed in white blood cells including leukocytes and macrophages (140). Upregulation of choline kinase in the activated macrophages may be the basic mechanism of FCH accumulation in inflammation (123, 141–143). Fibroblast activation protein (FAP) is overexpressed in activated fibroblasts that are present in the tumor stroma, fibrosis, and neo angiogenesis, which enables FAPI PET/CT to depict COVID-19 related pneumonia (144–146).

After COVID-19 vaccination, uptake in ALN was observed with ^68^Ga-DOTATATE (88, 147–149), ^68^Ga-DOTATOC (150, 151), ^18^F-fluciclovine (152, 153), ^68^Ga- or ^18^F-PSMA (88), ^18^F-rhPSMA-7.3 PET/CT (154), FCH (155–157), ^11^C-choline (100), ^18^F-florbetaben (158), and ^18^F-DOPA (88). Positive FDG and ^68^Ga-DOTATATE ALN uptake, respectively, was confirmed in 45 and 55% of the study cohort, which was a much higher incidence than that of ^68^Ga- and ^18^F-PSMA (0.3%) (88). Ah-Thiane et al. reported that FCH (40.7%) showed a much higher incidence of vaccine-related LN uptake than ^68^Ga-PSMA-11 (12.7%), and no patient showed vaccine-related LN uptake with ^18^F-FDOPA or ^68^Ga-DOTATOC PET/CT (159). In another report, FDG-positive ALNs were found in 10.4% of the cohort, whereas those with ^11^C-choline was 23.1% (100).

Intracellular immunoreactivity was significantly increased by SARS-CoV-2 in the presence of Aβ1–42, which is a strong indicator of Alzheimer’s disease with a high affinity for SARS-CoV-2 spike protein S1 subunit. Aβ1–42 significantly enhanced SARS-CoV-2 infectivity, whereas clearance of Aβ1–42 can be reduced during SARS-CoV-2 infection (160). Therefore ^18^F-florbetaben PET/CT is hypothesized to demonstrate immune-induced findings related to COVID-19 vaccination (158). Regarding the deltoid muscle and/or subcutaneous adipose tissues, PET uptake was reported with ^18^F-fluciclovine (152), ^18^F-florbetaben (158), FCH (156), ^11^C-choline (100), and ^68^Ga-DOTATATE (88) (Table 2). Nevertheless, the duration of PET imaging after COVID-19 vaccination differed among studies and the findings may not be limited to these PET tracers.

**TABLE 2:** COVID-19 related findings with non-FDG PET tracer.

COVID-19 related findings	Non-FDG PET tracer
COVID-19 pneumonia	^68^Ga-PSMA, ^18^F-PSMA-1007, ^18^F-PSMA, ^18^F-fluorocholine, ^68^Ga-DOTATATE, ^68^Ga-DOTANOC, ^68^Ga-FAPI-46, ^18^F-avb6-BP, ^82^Rb
Reactive lymph node with COVID-19 infection	^68^Ga-DOTANOC, FCH, ^68^Ga-PSMA
Reactive lymph node after COVID-19 vaccination	^68^Ga-DOTATATE, ^68^Ga-DOTATOC, ^18^F-fluciclovine, ^68^Ga- or ^18^F-PSMA, ^18^F-rhPSMA-7.3 PET/CT, ^18^F-fluorocholine, ^11^C-choline, ^18^F-florbetaben, ^18^F-DOPA
Deltoid muscle and/or subcutaneous adipose tissues	^18^F-fluciclovine, ^18^F-florbetaben, ^18^F-fluorocholine, ^11^C-choline, ^68^Ga-DOTATATE

Antoni developed the ^11^C-GW457427 PET tracer targeting the neutrophil elastase that exists in neutrophil granulocytes, involvement with which leads to severe lung inflammation in COVID-19 infection (161–163). ^11^C-GW457427 accumulation in COVID-19 opacities indicates high levels of neutrophil elastase. This author also used ^15^O-water to evaluate lung perfusion, and found that perfusion was severely reduced in COVID-19-infected lungs compared to healthy lung tissue (161).

^68^Ga-HZ20 and ^64^Cu-HZ20 were developed to visualize ACE2 expression in the human body. In a comparison of patients who had recovered from COVID-19 and volunteers, the COVID-19 recovered patients showed higher ^68^Ga-HZ20 uptake in most organs. Uptake in the oropharynx, nasal mucosa, and eyes (which are exposed to virus entry) and the lungs (which are the most affected by the virus) was similar or slightly higher in recovered patients than in healthy volunteers (164). A variety of NM tracers have potential for detecting and evaluating the range of complications that can be caused by COVID-19 (165, 166). In addition, several potential targets for PET imaging based on the pathophysiological features of COVID-19 have been introduced for the future management of COVID-19 patients (167).

## Impact of COVID-19 on oncology patients

Social trends toward refraining from hospital consultation and regularly scheduled hospital or clinic visits have been caused by restrictions in the general medical care system and people’s anxiety about contracting COVID-19. During the first wave of the pandemic in the UK (March to August 2020), an estimated 45% of people with potential cancer symptoms did not contact their doctor (168, 169). COVID-19 had a marked impact on cancer care, with 46% of patients experiencing a change in care, including treatment delay in 33% of patients and change of care location in 12%. The average duration of cancer-related care delay was greater than 4 weeks in 71.4% of clinic visits, 79.3% of laboratory testing or blood work, and 80.0% of imaging examinations (170). In the state of Victoria, Australia, it was estimated that approximately 2,500 cancer diagnoses were missed during the first 6 months of the pandemic (171). Based on a survey of 507,307 COVID-19 patients including 14,287 cancer patients, a higher risk of death (OR, 1.74), ICU stay (OR, 1.69), and hospitalization (OR, 1.19) was observed in patients with recent cancer treatment. In comparison, patients without recent cancer treatment had similar or better outcomes (mortality OR, 0.93; mechanical ventilation OR, 0.61) (172).

Based on a prospective cohort study conducted from March 2020 through July 2021 in the US for patients with any cancer diagnosis who were scheduled for treatment and contracted COVID-19, delay or discontinuation of anticancer drug, radiation treatment and surgical treatment occurred in 46, 47, and 71% of each cohort, respectively. The number of comorbidities, area of residence, and ethnicity were associated with delayed treatment (173). These findings raised strong concern that a large number of patients would present with more advanced cancer in the future (168).

The ONCOVIPET study reported the impact of the COVID-19 pandemic and national lockdown in Italy as a surrogate marker of the extent of cancer disease at FDG PET/CT staging. A comparison between 240 cancer patients in 2019 and 371 cancer patients in 2020 found a significant increase in the number of patients with advanced disease (rate, 1.56), nodal involvement (rate, 1.84) and metastasis (rate, 2.09) in 2020. Compared with cancer patients in 2019, those in 2020 had significantly greater nodal involvement (rate, 2.01) and metastasis (rate, 2.06). There was a significant increase in advanced disease in ML and lung cancer in 2020 compared to 2019, with significantly higher rates of LN involvement in lung cancer, gastrointestinal cancer, breast cancer, and ML; and significantly higher rates of metastasis in lung cancer (174).

A study conducted in Tokyo, Japan, compared trends in initial staging and restaging with FDG PET/CT for lung cancer, esophageal cancer, colon cancer, and ML between the period approximately 2 years before declaration of the COVID-19 pandemic and during the pandemic. The COVID-19 pandemic influenced the number of cancer patients who underwent FDG PET/CT. There was a marked decrease in the number of cancer patients who underwent FDG PET/CT between March 2020 and February 2021, followed by a recovery between March 2021 and December 2021. There was no significant difference between pre- and post-COVID-19 pandemic in terms of the initial stage of cancer, but diagnoses of Stage IV ML and Stage II esophageal cancer were more frequent after declaration of the pandemic. Initial staging of ML, lung cancer, and esophageal cancer revealed more advanced stages after declaration of the pandemic compared with March 2020 to February 2021. The duration between the last vaccination and FDG PET/CT showed several peaks in patient numbers for restaging, but there was no remarkable peak in those for initial staging. Although it has been recommended to avoid FDG PET/CT for at least 6 weeks after vaccination, patient status had been given priority in the decision whether or not to perform FDG PET/CT (175).

Spontaneous regression of lesions after COVID-19 infection and vaccination has been reported in several types of malignancy (lung cancer, ML, renal carcinoma, and colorectal carcinoma) (176–181). In patients with follicular lymphoma, FDG PET/CT showed an increase in the size of FDG-avid lesions after COVID-19 infection and later showed complete remission (176). Regarding the background of regression, there was a reduction of Ki67 positive cells and robust tumor immune cell infiltration such as higher fractions of T cells (especially CD8 + T cells), granzyme B + cells, B cells, and dendritic cells (182). The COVID-19 vaccine can induce both virus-specific antibodies and T-cell responses (183). As well as inducing autoimmunity, COVID-19 vaccines also enhance antitumor responses resulting from overstimulation of the immune system. In another hypothesis, viral proteins mimic human molecules such as molecular chaperones/heat shock proteins, which elicits immunity not only against themselves but also against the human proteins expressed on tumor cells (184, 185).

## Impact of COVID-19 on cardiology

There was a reduction in cardiac imaging volume in the early months of the pandemic (March and April 2020) compared to those in March 2019. Among the cardiac imaging tests, the smallest reduction was for stress PET, which declined by 58% in the US and by 51% in other countries. FDG-PET for the diagnosis of cardiac infection declined by 80% in the US but by 58% in other countries (186). The same trend was reported for the volume of PET examinations, which decreased worldwide by 34% in March 2020 and 56% in April 2020 compared to March 2019. Stress SPECT was more strongly affected, with reductions of 42 and 74%, respectively (187). In Oceania, cardiac PET and stress cardiac magnetic resonance (CMR) were the only modalities that did not show a significant reduction between March 2019 and April 2020 May. Cardiac PET has been suggested as an alternative to transesophageal echocardiography for detection of endocarditis in high-risk patients, but the number of examinations did not increase during this period, presumably due to the lack of reimbursement (188).

Compared with 2019, there was a reduction in stress PET examinations of 42% in Europe and 59% in the rest of the world in April 2020, and a reduction in PET performed for infection of 53% in Europe and 71% in the rest of the world in March 2020. In April 2020, lower gross domestic product and increasing COVID-19 deaths were independent predictors of the reduction in the volume of cardiac imaging procedures (189).

Because the COVID-19 virus spreads via aerosol droplets, pharmacological stress is preferred over exercise stress testing for MPI. Even in pharmacological stress testing, extra-long tubing was recommended to maximize the distance between staff and the patient (190, 191). ^82^Rb or ^13^N-ammonia myocardial perfusion PET are preferable to myocardial perfusion scintigraphy due to the shorter imaging time of only 30–45 min for the complete rest-stress study acquisition, which is shorter than for cardiac SPECT (190, 192). In addition, the CT attenuation scans can be reviewed during the PET examination to screen for the findings of COVID-19 pneumonitis (193).

## Positron emission tomography imaging of myocardial complications related to COVID-19

COVID-19 infection can trigger cardiac complications such as acute coronary syndrome (ACS), heart failure, cardiogenic shock, arrhythmias, and myocarditis (194, 195). In patients with COVID-19, the pooled incidence of acute myocardial infarction (AMI), heart failure, arrhythmia, cardiac arrest, and ACS was 21, 14, 16, 3.46, and 1.3%, respectively. Patients with severe disease were at higher risk of AMI (RR = 5.27) and shock (OR = 20.18) compared with non-severe cases (196). In addition, myocarditis caused by COVID-19 infection resulted in mortality of approximately 7% (197).

Vaccine-associated myocarditis was less severe than other causes of myocarditis, although CMR showed a similar pattern of myocardial injury (such as frequent LGE occurrence) in the subepicardium at the basal inferolateral wall (198).

Hanneman et al. (199) assessed myocardial injury in patients who had recovered from COVID-19 (mean 67 ± 16 days from the diagnosis of COVID-19) using FDG PET/MRI. Focal or focal on diffuse patterns of FDG uptake were considered positive findings, and the most involved myocardial segment was the mid-inferolateral wall, followed by the basal inferolateral, basal antero-septum, mid-inferoseptum, and mid-inferior wall. Focal FDG uptake was observed most frequently in patients with hypertension and cardiac symptoms.

Regarding the CMR image findings, there was a greater association of late gadolinium enhancement (LGE); higher regional T2, T1, and ECV; lower left ventricular ejection fraction (LVEF); and worse global longitudinal strain and global circumferential strain with focal FDG uptake than without focal FDG uptake. Patients with focal FDG uptake had improved cardiac function. The presence of inflammation on MRI at short-term follow-up appears to indicate that myocardial inflammation after COVID-19 resolves without treatment (199).

In their survey of 105 patients who had recovered from COVID-19, Sarıçam et al. (200) found increased NT-proBNP levels, low serum nitric oxide levels, and increased FDG uptake on cardiac PET in post-acute COVID syndrome. Cardiac PET could replace or be added to CMR to detect subtle subacute/chronic myocarditis (200).

The presence of myocardial inflammation/edema on CMR or PET can be useful in distinguishing acute from chronic left ventricular dysfunction. In patients with known coronary artery disease (CAD) with suspected low-risk ACS, vasodilator stress MRI or radionuclide myocardial perfusion imaging can be considered, especially PET if available. Patients with new left ventricle systolic dysfunction should be investigated for the presence of underlying CAD. For those without evidence of CAD, CMR or PET can provide important insights into the etiology of myocardial dysfunction (201, 202).

In another study, inflammatory status at the presumed peak of the inflammatory phase was assessed by FDG PET/CT in 13 non-critically ill inpatients with COVID-19 (42). Patients were enrolled prospectively and underwent an FDG PET/CT examination within days 6–14 after onset of symptoms. Only one of these patients had significant physiological myocardial FDG uptake, even though there had been no intervention to suppress physiologic myocardial glucose metabolism prior to scanning; although normally, substantial myocardial tracer uptake might have been expected in most individuals. The authors suggested that the myocardial metabolic pathway may disfavor glycolysis during COVID-19 infection, perhaps due to a loss of sympathetic tone that would otherwise promote myocardial FDG uptake (203).

Astley et al. (204) evaluated myocardial blood flow by ^13^N-ammonia PET/CT in children with multisystem inflammatory syndrome that developed after acute respiratory syndrome caused by COVID-19. They found severe perfusion defect in 2/5 cases, with decreased myocardial flow reserve in the slightly dilated left ventricular cavity, even though the coronary arteries appeared normal in all patients. The authors assumed that the abnormal myocardial flow reserve was caused by coronary microvascular dysfunction resulting from vasomotor dysregulation or endothelial dysfunction of the small coronary arterioles (204).

Myocarditis after COVID-19 vaccination has been reported predominantly in younger subjects and after the second dose of mRNA vaccines (194, 205). The mechanisms of COVID-19 mRNA vaccine leading to myocarditis are autoimmune and autoinflammatory responses (206, 207), autoantibody generation (208), and molecular mimicry between the viral spike protein and self-antigens (209, 210). Myocarditis was depicted by FDG PET/MRI on day 5 after a second COVID-19 vaccination in a patient who had undergone preparation of a low carbohydrate diet followed by 12-h fasting (211). Focal FDG uptakes on the wall of the left ventricle were concordant with LGE of MRI. Boursier reported ^68^Ga-DOTATOC PET imaging of myocarditis that occurred 2–3 days after the second dose of an mRNA COVID-19 vaccine (212). It is estimated that inflammatory cells such as lymphocytes, macrophages, and activated monocytes overexpress somatostatin receptors (213), which may be supported by the COVID-19 unrelated myocarditis depicted by somatostatin receptor PET and scintigraphy (214, 215).

## Conclusion

During the COVID-19 pandemic, the role expected of the NM department was to continue to provide essential and critical services. Although no absolute necessity has emerged for FDG PET/CT in the diagnosis or management of COVID-19, it may nevertheless offer insight into the background of patients who show atypical symptoms after COVID-19 infection and long-term effects as called “Long COVID.” For further insight, it is important to follow how COVID-19 impacts patients with malignancy and/or cardiac disease.

## Author contributions

The author confirms being the sole contributor of this work and has approved it for publication.
